# A novel clinical tool to classify facioscapulohumeral muscular dystrophy phenotypes

**DOI:** 10.1007/s00415-016-8123-2

**Published:** 2016-04-28

**Authors:** Giulia Ricci, Lucia Ruggiero, Liliana Vercelli, Francesco Sera, Ana Nikolic, Monica Govi, Fabiano Mele, Jessica Daolio, Corrado Angelini, Giovanni Antonini, Angela Berardinelli, Elisabetta Bucci, Michelangelo Cao, Maria Chiara D’Amico, Grazia D’Angelo, Antonio Di Muzio, Massimiliano Filosto, Lorenzo Maggi, Maurizio Moggio, Tiziana Mongini, Lucia Morandi, Elena Pegoraro, Carmelo Rodolico, Lucio Santoro, Gabriele Siciliano, Giuliano Tomelleri, Luisa Villa, Rossella Tupler

**Affiliations:** Department of Life Sciences, University of Modena and Reggio Emilia, Modena, Italy; Department of Clinical and Experimental Medicine, Neurological Clinic, University of Pisa, Pisa, Italy; Department of Neurosciences, Reproductive and Odontostomatological Sciences, University Federico II of Naples, Naples, Italy; Department of Neuroscience, Center for Neuromuscular Diseases, University of Turin, Turin, Italy; MRC Centre of Epidemiology for Child Health, UCL Institute of Child Health, London, UK; IRCCS San Camillo, Venice, Italy; Department of Neuroscience, Mental Health and Sensory Organs, S. Andrea Hospital, University of Rome “Sapienza”, Rome, Italy; Unit of Child Neurology and Psychiatry, IRCCS “C. Mondino” Foundation, Pavia, Italy; Department of Neurosciences, University of Padua, Padua, Italy; Center for Neuromuscular Disease, CeSI, University “G. D’Annunzio”, Chieti, Italy; Department of Neurorehabilitation, IRCCS Institute Eugenio Medea, Bosisio Parini, Italy; Neurology Clinic, ‘‘Spedali Civili’’ Hospital, Brescia, Italy; IRCCS Foundation, C. Besta Neurological Institute, Milan, Italy; Neuromuscular Unit, Fondazione IRCCS Ca’ Granda Ospedale Maggiore Policlinico, Dino Ferrari Center, University of Milan, Milan, Italy; Department of Clinical and Experimental Medicine, University of Messina, Messina, Italy; Department of Neurological, Neuropsychological, Morphological and Movement Sciences, University of Verona, Verona, Italy; Department of Molecular Cell and Cancer Biology, University of Massachusetts Medical School, Worcester, USA

**Keywords:** FSHD, Clinical phenotype, Diagnostic criteria, Disease registry, Disease classification

## Abstract

Based on the 7-year experience of the Italian Clinical Network for FSHD, we revised the FSHD clinical form to describe, in a harmonized manner, the phenotypic spectrum observed in FSHD. The new Comprehensive Clinical Evaluation Form (CCEF) defines various clinical categories by the combination of different features. The inter-rater reproducibility of the CCEF was assessed between two examiners using kappa statistics by evaluating 56 subjects carrying the molecular marker used for FSHD diagnosis. The CCEF classifies: (1) subjects presenting facial and scapular girdle muscle weakness typical of FSHD (category A, subcategories A1–A3), (2) subjects with muscle weakness limited to scapular girdle or facial muscles (category B subcategories B1, B2), (3) asymptomatic/healthy subjects (category C, subcategories C1, C2), (4) subjects with myopathic phenotype presenting clinical features not consistent with FSHD canonical phenotype (D, subcategories D1, D2). The inter-rater reliability study showed an excellent concordance of the final four CCEF categories with a *κ* equal to 0.90; 95 % CI (0.71; 0.97). Absolute agreement was observed for categories C and D, an excellent agreement for categories A [*κ* = 0.88; 95 % CI (0.75; 1.00)], and a good agreement for categories B [*κ* = 0.79; 95 % CI (0.57; 1.00)]. The CCEF supports the harmonized phenotypic classification of patients and families. The categories outlined by the CCEF may assist diagnosis, genetic counseling and natural history studies. Furthermore, the CCEF categories could support selection of patients in randomized clinical trials. This precise categorization might also promote the search of genetic factor(s) contributing to the phenotypic spectrum of disease.

**Electronic supplementary material:**

The online version of this article (doi:10.1007/s00415-016-8123-2) contains supplementary material, which is available to authorized users.

## Introduction

Facioscapulohumeral muscular dystrophy (FSHD) is one of the most common forms of hereditary myopathy [1]. The classical FSHD phenotype is rather distinctive, characterized by a progressive asymmetric facial, shoulder girdle and pectoral muscle weakness and atrophy, with a descending progression to involve the distal lower extremity muscles before affecting the hip girdle muscles [2]. However, a wide variability of clinical expression has been extensively documented [3].

At present, two genetically distinct disease subtypes, FSHD1 and FSHD2 are described. The molecular defect associated with FSHD1 resides in a stretch of tandemly arrayed 3.3 kb repetitive elements, named D4Z4, ranging from 11 to 150 repeat units in healthy subjects [4]. Alleles with 8 or fewer D4Z4 repeats on chromosome 4q have been found in the majority of FSHD patients. FSHD2 patients carry D4Z4 alleles of size at the lower end of the general healthy population range size [5]. In these patients, the disease is associated with heterozygous dominant mutations in the *SMCHD1* gene [6].

However, D4Z4 alleles in the size-range of FSHD1 patients (4–8 units, 20–35 kb EcoRI alleles) are carried by 3 % of healthy control population [7–9]. Thus, a D4Z4 allele of reduced size may be permissive but it is not sufficient to develop autosomal dominant disease. Consistently, in FSHD families, we found that almost 25 % of FSHD heterozygotes older than 55 years were asymptomatic [10]. Moreover, there are families in which the disease appears only in one generation or in a single subject [8, 10] with no other relatives with signs of disease. Besides, several reports describe atypical phenotypes in carriers of a D4Z4 reduced allele (DRA) [11].

Collectively, the extensive use of DNA analysis in FSHD has revealed an unanticipated complexity without a straightforward correlation between the clinical phenotype and molecular variations. Incomplete penetrance and wide clinical variability argue for the role of modifying loci or epigenetic mechanisms influencing the clinical expression of disease. This clinical and genetic variability, which is observed also in other hereditary neuromuscular diseases, represents an obstacle for the interpretation of clinical data, for genotype-phenotype correlations, appropriate genetic counseling and for the definition of a minimal dataset necessary for the stratification of patients eligible for therapeutic trials. Therefore, to formulate optimal diagnostic criteria, molecular analysis must be associated with standardized and harmonized clinical evaluation.

Here, in light of our 7-year experience, we present the FSHD Comprehensive Clinical Evaluation Form (CCEF), a modified version of the original FSHD Clinical Form [12] for the detailed description of all phenotypic features detected in FSHD patients and families.

## Methods

### Study design

**Fig. 1:**
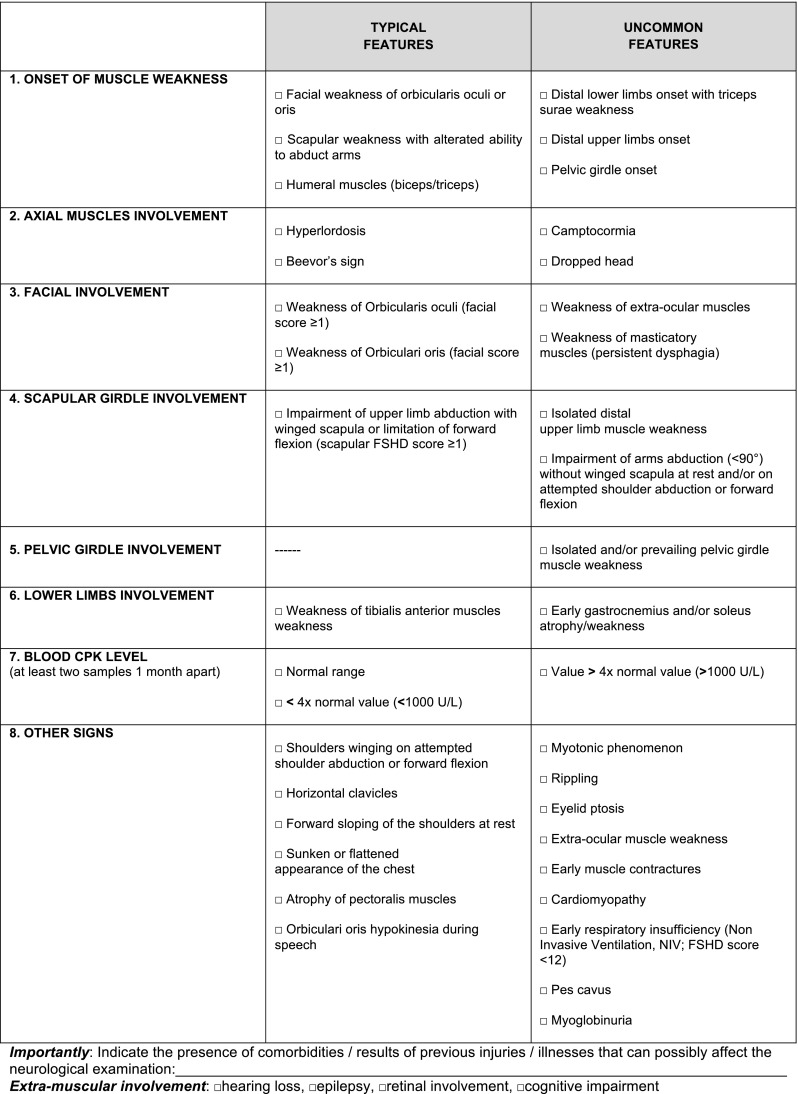
CCEF Section 3: Clinical Diagnostic Form

**Fig. 2:**
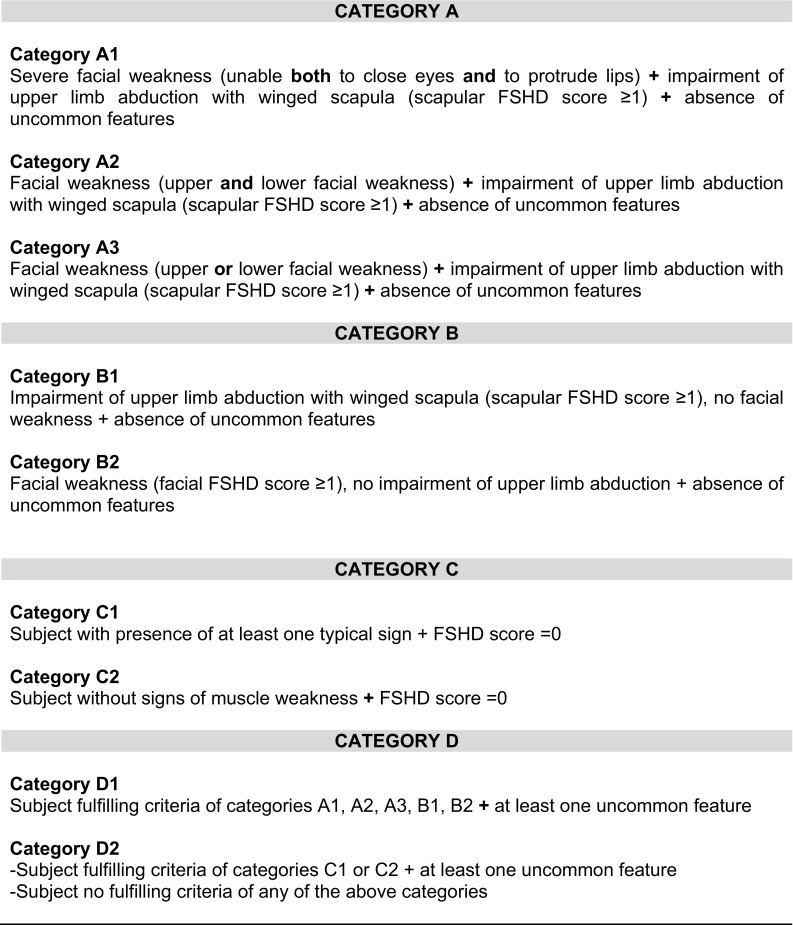
CCEF Section 4: Clinical Categories

Through the systematic use of the FSHD Clinical Form [10, 12, 13] we recognized that it assesses the severity of motor impairment by translating disability into a number (*FSHD Evaluation Scale*, CCEF Section 2, Supplementary Figure 1), but it does not capture clinical features that may describe various phenotypes. To overcome this limitation, we integrated several items including typical and atypical features on the basis of published reports describing the clinical phenotypes observed in carriers of a DRA (reviewed in [11]). Typical and atypical clinical features were combined in the new CCEF, which includes the *Evaluation Form* (CCEF Section 1, Supplementary Figure 1), the *FSHD Evaluation Scale* (CCEF Section 2, Supplementary Figure 1), the *Clinical Diagnostic Form* (CCEF Section 3, Fig. 1), and the *Clinical Categories* (CCEF Section 4, Fig. 2). The integral CCEF can be downloaded as Supplementary Figure 1 and at http://www.fshd.it. The definition and the validation of the CCEF were performed in two steps. We first recruited 106 subjects carrying a DRA with 1–9 units (11–38 kb) to test the clinical application of this new tool. The recruitment was based on 452 subjects examined by the Italian Clinical Network for FSHD (ICNF) in 2-year time-window (2008–2009). Subjects were summoned by consecutive phone calls following the order of the previous recruitment. We called those near the clinical centers of Modena, Turin and Naples. The latter choice was made to avoid people a long-distance trip. We organized three meetings dividing the 106 available subjects into three groups on the basis of their geographic location (Northern, Central and Southern Italy). Twelve experienced clinicians of the ICNF were selected according to their geographic location, so that four neurologists examined patients from each one of the three groups. The four selected neurologists used the CCEF to evaluate each subject of a single group independently. The results of this first round of clinical applications were discussed in a subsequent meeting. We revised the emerged critical points, i.e. some difficulties in establishing mild facial weakness, and approved the final version of the CCEF (Supplementary Figure 1). Then, in a second round, the inter-rater reliability in assigning patients to different phenotypic categories using the new CCEF was tested. Two clinicians, selected by drawing lots, examined additional 56 subjects (Supplementary Table 1) recruited from the cohort of 452 subjects as described above. The two clinicians administered the functional motor evaluation test of the *Evaluation Form* (Supplementary Figure 1, Section 1, parts b and c) to each subject and calculated the FSHD clinical score on the basis of the *FSHD Evaluation Scale*, previously validated [12]. Then, the two clinicians completed the *Clinical Diagnostic Form* (CCEF Section 3, Fig. 1) and assigned each subjects to one of the nine clinical subcategories (CCEF Section 4, Fig. 2) independently. A tutorial for the clinical assessment is available at http://www.fshd.it. It takes 20 min to collect clinical information and complete the neurologic evaluation.

The subject recruitment was approved by the Ethics Committee of Modena and all the participating centers. Signed informed consent from patients was obtained before inclusion in the study.

### Statistical analysis

The inter-observer reproducibility between the two examiners respect to the four and nine CCEF categories was assessed using the kappa statistics [14]. *κ* value scores are interpreted as follows: *κ* value 1.0 = perfect agreement; *κ* value ≥0.75 < 1.0 = excellent; *κ* value >0.40 < 0.75 = good; *κ* value ≤0.40 = poor. The 95 % confidence intervals of kappa statistics were calculated using the (biased corrected) bootstrap resampling method [15].

## Results

### A tool to describe clinical variability

The CCEF consists of four sections. The first section, the *Evaluation Form* (Section 1, Supplementary Figure 1), investigates the subject’s clinical history (part a), evaluates the patient’s disability (part b) and assesses muscle segmental involvement using the Medical Research Council (MRC) scale (part c). The other sections include the *FSHD Evaluation Scale* (Section 2, Supplementary Figure 1), the *Clinical Diagnostic Form* (Section 3, Fig. 1) and the *Clinical Categories* (Section 4, Fig. 2).

Several items are examined in the *Evaluation Form* section.

#### Family history

Questions such as “did/does any of your relatives have a posture like yours?”, “was any of your relatives sleeping with half-open eyes?” are asked to identify subjects with possible muscle weakness suggestive of FSHD.

#### Evaluation of age at onset

To obtain a more objective evaluation of age at onset and the type of muscle initially affected, we introduced specific questions, such as “have your relatives ever noticed that you were sleeping with half-open eyes?”, “when have you noticed the appearance of winged scapula?”, “have you ever noticed thinness of upper arms or a dropped shoulder?”, “have you ever noticed asymmetry of the mouth or smile when looking in a mirror or in past photographs from childhood?”.

#### Functional motor evaluation

For a precise description of the distribution of muscle weakness, the CCEF evaluates: (a) the presence of widened palpebral fissures; *orbicular oris* weakness, horizontal smile; inability to protrude lips, to puff out cheeks, to close eyes and bury the eyelashes (facial weakness); (b) the maximum degree in abducting arms (scapular girdle weakness); (c) the ability to climb 4 stair-steps, to stand up from a chair, to rise from the floor, to walk (pelvic girdle weakness); (d) the ability to walk on tiptoes and/or heels (distal legs weakness); (e) the presence of Beevor’s sign (abdominal muscles weakness).

#### Evaluation of segmental muscle strength by MRC scale

Fourteen muscle groups are examined. Neck extensors are evaluated as single muscle group; external-rotator muscles of upper limb, triceps, biceps, common finger extensors, wrist extensors, long fingers flexors, wrist flexors, gluteus maximum, iliopsoas, quadriceps, biceps femoris, triceps surae, tibialis anterior are evaluated on both sides.

#### Annotation of typical signs

Shoulders with symmetric/asymmetric winging on attempted shoulder abduction or forward flexion, straight clavicles, forward sloping of shoulders at rest, axillary creases reflecting pectoral muscle wasting, sunken or flattened appearance of the chest, “poly-hill sign” with neck, shoulders and arms observed from behind in fullest possible abduction (70°–90°), with external rotation of the shoulders, hyperlordosis.

#### Annotation of atypical signs

Palpebral ptosis [2], myotonic phenomenon [16], muscle rippling [17], weakness of extra-ocular [2], masticatory, pharyngeal and lingual muscles [2, 18], bent spine syndrome [19], early contractures [2], *pes cavus* [20], dropped head, myoglobinuria and persistently high CK values above the level of 1000 U/L are [2] considered atypical signs. The presence of cardiomyopathy and a respiratory restrictive insufficiency at onset or in subjects still walking (FSHD score <12) is also considered an atypical sign [2, 21].

The *Evaluation Form* allows completing the *FSHD Evaluation Scale* to calculate the FSHD clinical score (Section 2, Supplementary Figure 1) [12]. The score considers the regional distribution of muscle weakness and the functionality of: (I) facial muscles (scored from 0 to 2); (II) scapular girdle muscles (scored from 0 to 3); (III) upper limb muscles (scored from 0 to 2); (IV) leg muscles (scored from 0 to 2); (V) pelvic girdle muscles (scored from 0 to 5); and (VI) abdominal muscles (scored from 0 to 1). Overall, the total FSHD score ranges from 0 to 15 and numerically defines the clinical severity of the motor impairment [10, 12, 13].

All sections of CCEF are used for the assessment and the classification of a patient. Based on the distribution of muscle weakness, scored by the *FSHD Evaluation Scale*, and the combination of the clinical features suggestive or not of FSHD, summarized in the *Clinical Diagnostic Form* (CCEF Section 3, Fig. 1), it is possible to assign patients to different phenotypic categories (CCEF Section 4, Fig. 2). In particular, we assigned (1) subjects with typical FSHD presenting facial and scapular girdle muscle weakness in category A; (2) subjects with muscle weakness limited to facial or scapular girdle muscles in category B; (3) asymptomatic subjects without motor impairment in category C; (4) subjects with myopathic phenotype presenting other anomalous clinical features not consistent with FSHD in category D.

**Fig. 3:**
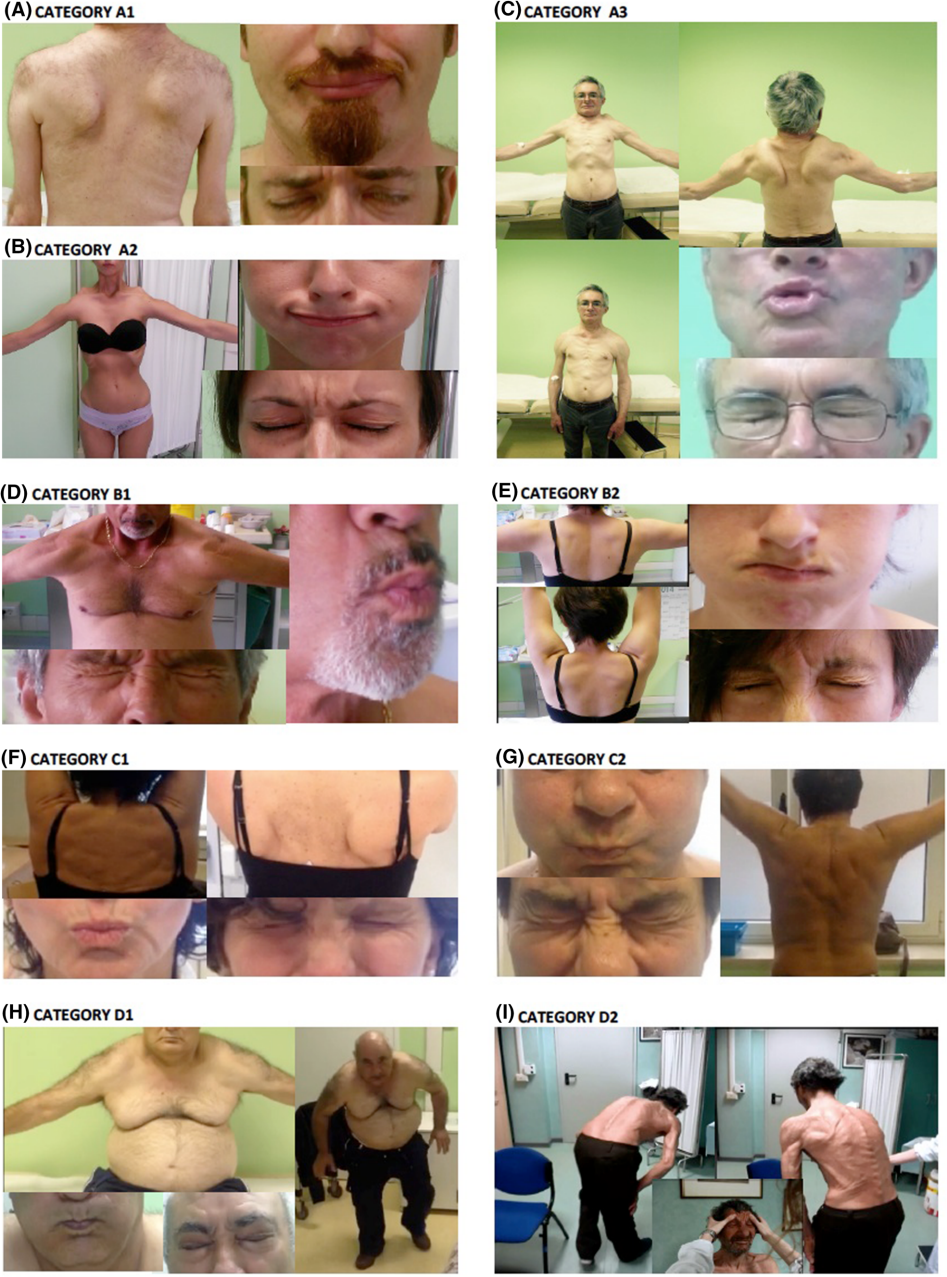
Examples of clinical categories: case reports. **a** Category A1: male, 38-year old, showing severe upper and lower facial weakness (unable to close both eyelids completely, puff cheeks and protrude lips), and impairment of upper limb abduction with winged scapula. **b** Category A2: female, 31-year old, with moderate upper (partial ability to close eyes, without the presence of widened palpebral fissures) and lower facial weakness (partial ability to puff out cheeks), impairment of upper limb abduction with winged scapula. **c** Category A3: male, 60-year old, with moderate lower facial weakness (partial ability to protrude lips), impairment of upper limb abduction with winged scapula. **d** Category B1: male, 66-year old, with impairment of upper limb abduction with winged scapula, no facial weakness. **e** Category B2: female, 34-year old, with moderate lower facial weakness (partial ability to puff out cheeks and to protrude lips), no scapular weakness. **f** Category C1: female, 55-year old, presenting asymmetric scapular winging on forward flexion without motor impairment (FSHD score 0). **g** Category C2: male, 56-year old, without motor impairment or other FSHD typical signs of muscle atrophy/weakness (FSHD score 0). **h** Category D1: male, 66-year old: onset after 50 age at shoulder girdle, without facial motor impairment and “bent spine”. **i** Category D2: male, 75-year old, with isolated bent spine syndrome, without signs suggestive of FSHD

Moreover, in view of our experience on FSHD phenotypes accrued through the past years in INRF [10, 13], we further described additional variants within each category (Fig. 2). Patients with typical phenotype were classified in three subcategories (A1–A3), on the basis of the severity of facial involvement, which seems to discriminate some classical phenotypes (Fig. 3a–c). This is because, we observed that some infantile forms or more severe phenotypes [13] are characterized by an early and prominent weakness of *orbicularis oculi* and *oris* with facial diplegia and dysartria. Thus, these patients were defined as category A1 to distinguish them from the vast majority of patients in which we observed a milder facial involvement (categories A2 and A3). This distinction should facilitate the identification of a specific clinical group deserving *ad hoc* studies.

Incomplete FSHD phenotype, not presenting a coexisting involvement of facial and scapular girdle muscles without other uncommon features, are considered category B1 or B2 (Fig. 3d, e). We identified these categories because, for instance, an isolated scapular girdle muscle weakness can be observed in FSHD relatives, but it can be also related to other myopathic disorders or nerve injuries.

Category D comprises myopathic subjects presenting some FSHD features in association with other uncommon characteristics suggestive of a possible comorbidity (D1) or patients that do not fulfill the diagnostic criteria for FSHD and can be affected by an alternative disease (D2) (Fig. 3h, i). Atypical features were chosen based on evidences from the literature [11]. This category may facilitate the discovery of factors that contribute to the disease expression or identify those subjects who are wrongly considered FSHD because of a diagnostic bias due to the random finding of DRA.

Finally, we decided to further differentiate non penetrant carriers: the asymptomatic subjects without motor impairment that present minor signs suggestive of FSHD (“typical features-other signs” Fig. 1) are described as category C1, whereas category C2 includes subjects with a neurologic examination completely normal (Fig. 3f, g). This distinction might be of particular importance for studying the natural history of disease (i.e. subjects described as C1 might develop clinical FSHD later or remain asymptomatic).

Overall, the categories we generated aim at describing different phenotypes thus capturing clinical diversity, regardless of the severity of motor impairment, otherwise reported as FSHD score.

### Inter-rater reliability of phenotype subgroups

The characteristics of the 56 FSHD patients enrolled in the inter-rater reliability study are shown in Supplementary Table 1. The sample is almost balanced by sex, 34 % aged less than 40 years, 12.5 % had an FSHD score higher than 10, all but three carried a DRA with 8 or fewer repeats (p13E−11 EcoRI fragments ≤35 kb).

**Table 1:** Agreement between Observer 1 and Observer 2 with respect to the nine CCEF categories classification

	CCEF categories	Observer 2									
		A1	A2	A3	B1	B2	C1	C2	D1	D2	Total
Observer 1	A1	6	2	0	0	0	0	0	0	0	8
	A2	1	18	2	0	0	0	0	0	0	21
	A3	0	2	4	2	0	0	0	0	0	8
	B1	0	0	1	5	0	0	0	0	0	6
	B2	0	0	0	0	2	0	0	0	0	2
	C1	0	0	0	0	0	2	0	0	0	2
	C2	0	0	0	0	0	1	4	0	0	5
	D1	0	0	0	0	0	0	0	2	0	2
	D2	0	0	0	0	0	0	0	0	2	2
	Total	7	22	7	7	2	3	4	2	2	56

*κ* = 0.75; 95 % CI (0.57; 0.87)

The concordance between the clinical assessments performed by the two neurologists was evaluated for the nine CCEF categories described in Fig. 2. As shown in Table 1, a good/excellent agreement [*κ* = 0.75; 95 % CI (0.57; 0.87)] was observed using the nine CCEF classifications. The overall kappa statistic combines the reliability of the nine categories with a perfect agreement observed for categories B2, C2, D1, D2; a good/excellent agreement for categories A1, A2, B1 and C2, and a good agreement observed for the category A3. The results of the concordance of the final four CCEF categories are presented in Table 2. As expected, the reliability increased with a *κ* equal to 0.90; 95 % CI (0.71; 0.97). A perfect agreement was observed for categories C and D, an excellent agreement for categories A [*κ* = 0.88; 95 % CI (0.75; 1.00)], and a good agreement for categories B [*κ* = 0.79; 95 % CI (0.57; 1.00)]. A lower level of *κ*, when compared with values obtained for each subcategory, is due to the increased number of categories taken into account in the final score and reflects the sensitivity of the test.

**Table 2:** Agreement between Observer 1 and Observer 2 with respect to the fourth CCEF categories classification

	CCEF categories	Observer 2				
		A	B	C	D	Total
Observer 1	A	35	2	0	0	37
	B	1	7	0	0	8
	C	0	0	7	0	7
	D	0	0	0	4	4
	Total	36	9	7	4	56

*κ* = 0.90; 95 % CI (0.71; 0.97)

## Discussion

The recently published Guidelines on FSHD of the American Academy of Neurology [22] represent an attempt toward the formulation of optimal standards of diagnosis and care for patients. In these recent Guidelines on FSHD, a relevant diagnostic significance is attributed to the detection of D4Z4 alleles associated with the 4qA polymorphism regardless of the phenotypic features. However, large-scale genotype-phenotype studies have revealed incomplete penetrance and wide variable expressivity in FSHD [8–11, 23] supporting the role of modifying loci or epigenetic mechanisms influencing the clinical expression of disease [5, 6]. Moreover, the FSHD molecular signature has a frequency of 1.3 % [7], which decreases the specificity of the molecular testing for FSHD. So, in our opinion, diagnosis of FSHD must be supported by the harmonized description of the observed clinical phenotypes and the family history.

Nowadays, studies suggest the role of epigenetic modifiers in FSHD onset and expression, including the level of 4q35 methylation and/or mutations in *SMCHD1* gene [5, 24]. Besides, a vast number of reports describe subjects with peculiar/atypical phenotypes carrying a DRA and suggest that mutations in other genes, i.e. gene associated with other neuromuscular diseases, might contribute to disease phenotype [11]. This genetic heterogeneity requires the harmonized classification of clinical phenotypes among patients and within families to serve clinical practice. In FSHD, intra-familial clinical variability is one of the most relevant challenges affecting clinical practice and genetic counseling. Our work shows that the CCEF is an easy clinical tool useful to capture various phenotypes from classic FSHD to individuals with incomplete phenotype, or asymptomatic carriers as well as subjects with atypical signs for which alternative diagnoses may be supposed. The choice of the nine categories responds to the necessity of describing the wide clinical spectrum of FSHD patients and their relatives with a simple and direct approach. Notably, the CCEF collects several items regarding anamnestic data, including onset, disease progression, distribution and degree of motor impairment (measured as the *FSHD Evaluation Scale*).

**Fig. 4:**
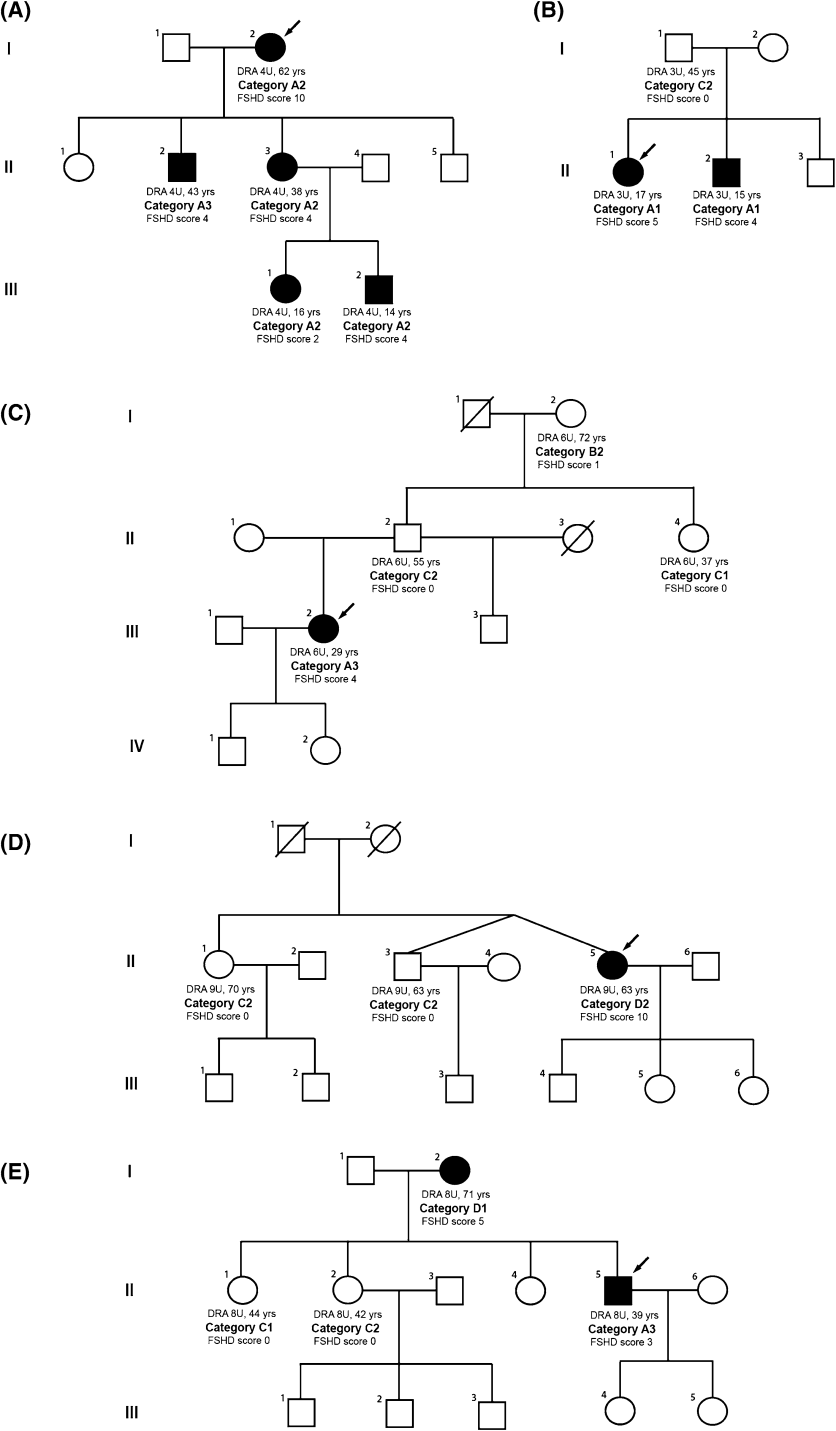
Clinical characterization of families in which a DRA segregates. Five families are presented. For each subject carrying a 4qA-type DRA on a permissive haplotype, age at evaluation, size of the DRA, clinical category and FSHD score are reported

By applying the CCEF, it will be possible to quickly classify families on the basis of the harmonized description of genotypes and phenotypes. This classification will support genetic counseling taking into account disease penetrance and expression within a single family. Figure 4 shows some examples. Figure 4a displays a family with the canonical autosomal dominant pattern of inheritance. The disease is present in all three generations and all subjects, carrying a DRA, display facial and scapular girdle weakness typical of FSHD, categories A2 and A3. Figure 4b shows a family in which two sibs are severely affected (A1) whereas the father carrying the same 3U DRA (no somatic mosaicism of the DRA was detected) is healthy (C2). Figure 4c presents a four-generation pedigree in which a single 29-year-old subject, III.2, developed mild weakness of *orbicularis oris* and weakness of scapular girdle muscle (category A3). She carries a 6U DRA inherited by her healthy 55-year-old father, II.2 (category C2). The paternal 37-year-old aunt, carrying the 6U DRA, is asymptomatic with non-specific signs as horizontal clavicles and axillary creases (category C1) and the paternal 72-year-old grandmother, I.2, carrying the 6U DRA, presents only incomplete and mild weakness of facial muscle (category B2). Figure 4d describes a family with a single patient presenting severe myopathy with atypical phenotype (D2). The 63-year-old proband carries a DRA with 9 units as do the twin brother and the 70-year-old sister, both healthy (C2). Finally, Fig. 4e displays a family that may mimic an autosomal dominant inheritance. The proband (II.5), carrying a DRA, presents a typical FSHD phenotype (A3). His mother (I.2) carries the same DRA, but she displays an atypical phenotype (D1) without the facial muscle involvement, and with an early and predominant involvement of the pelvic girdle probably related to old age. Instead, his two older sisters (II.1 and II.2) are asymptomatic carriers. In our opinion, all these unexpected distribution of clinical phenotypes require particular attention in evaluating the risk of disease onset and expression, and the possible contribution of genetic modifiers. Indeed, the systematic application of the CCEF might support physicians in the identification of these critical families that might be suitable for further investigations and promote the understanding of disease pathophysiology.

Moreover, using the CCEF, it is possible to obtain the longitudinal trajectory of disease progression for each patient and describe the disease’s natural history, including the follow-up of non-manifesting carriers.

Overall, the CCEF is a flexible tool that can assist novel strategies to study the etiology of rare diseases. It can support a catalog of the phenotypes observed among and within families facilitating the phenotypic stratification of FSHD patients, the search of genetic modifiers, and studies on the natural history of disease. Finally, the harmonized clinical classification of subjects is fundamental for the stratification of patients eligible for clinical trials. In this perspective, the CCEF can be an instrument for observational studies or randomized clinical trials.

## Electronic supplementary material

Below is the link to the electronic supplementary material.

Supplementary material 1 (PDF 221 kb)

Supplementary material 2 (PDF 51 kb)
