# Supplementary material for: A novel clinical tool to classify facioscapulohumeral muscular dystrophy phenotypes
Source: J Neurol. 2016 Apr 28;263:1204–14. doi: 10.1007/s00415-016-8123-2 (PMC4893383; doi:10.1007/s00415-016-8123-2)
Supplement: Supplementary file 1 — Supplementary material 1 (PDF 221 kb) [file 415_2016_8123_MOESM1_ESM.pdf]

Part A

Referring Hospital: ____________________________ Referring Physician: ________________________

Compilation Date: _ _/_ _ /_ _ _ _

Patient Initials: ____________________________ Patient code: _______________

Date of birth: _ _/_ _ /_ _ _ _ Sex: ○ M ○ F

Handedness: ○ Left ○ Right

Weight:__________kg

Height:__________cm

Family Ancestry - geographic origins:

Maternal: ____________________________ Mother Surname: _________________________

Paternal: ____________________________

Consanguinity: ○ Yes ○ No

Current profession: ___________________________ Since year: _ _ _ _

If you were previously employed:

Previous profession(s) :

____________________________ From year: _ _ _ _ to year _ _ _ _

____________________________ From year: _ _ _ _ to year _ _ _ _

____________________________ From year: _ _ _ _ to year _ _ _ _

Highest degree: ○ University degree ○ High-school diploma ○ Primary school diploma ○ None

(years of education: ________)

Clinical history

Previous evaluation in other center(s): ○ Yes ○ No If yes, centre: (1) ____________________

(2) ____________________

FSHD score at last clinical examination: _ _ Date: _ _/_ _ /_ _ _ _

Comorbidities:

Diabetes mellitus: ○ Yes ○ No ○ Not evaluated

If Yes, ○ type I ○ type II Age at diagnosis: _ _

Therapy, Drugs :

____________________ Dose: _ _ _ _ unit _ _ _ _ From year: _ _ _ _ to year _ _ _ _

____

____

Thyroid hormones alterations: ○ Yes ○ No ○ Not evaluated

If Yes, ○ hypothyroidism ○ hyperthyroidism Age at diagnosis: _ _

Therapy, Drugs :

____________________ Dose: _ _ _ _ unit _ _ _ _ From year: _ _ _ _ to year _ _ _ _

____

Hepatitis: ○ Yes ○ No ○ Not evaluated

If Yes, ○ HBV ○ HCV ○ Toxic Age at diagnosis: _ _

Therapy, Drugs :

____________________ Dose: _ _ _ _ unit _ _ _ _ From year: _ _ _ _ to year _ _ _ _

____

Diagnosis of cancer: ○ Yes ○ No

If yes, specify:____ Age at diagnosis: _ _

Therapy, Drugs :

____________________________ Dose: _ _ _ _ unit _ _ _ _ From year: _ _ _ _ to year _ _ _ _

____

Retinal vasculopathy: ○ Yes ○ No ○ Not evaluated

Sensorineural deafness: ○ Yes ○ No

Audiometry: ○ Alterated ○ Normal ○ Not performed

Epilepsy: ○ Yes ○ No

Cognitive impairment: ○ Yes ○ No

Other disease(s)

Other diseases ○ Yes ○ No

If yes, specify: ____

____ ___________________

____

Previous trauma: joint, bone fractures: ○ Yes ○ No If yes, specify site and age_ _

Drugs:

Statins: ○ Yes ○ No If yes, Type: ______________________________

Dose: _ _ _ _ unit _ _ _ _ From year: _ _ _ _ to year _ _ _ _

Others chronic treatments: ○ Yes ○ No

If yes

Drug: ______________________ Dose: __________________ From year: _______to year _______

____

____

Reproductive History:

Have you ever been pregnant? ○ Yes ○ No

Are you pregnant now? ○ Yes ○ No

How many times have you been pregnant: _ _

Spontaneous abortion: ○ Yes ○ No if yes, Number of spontaneous abortion: _ _

How many vaginal deliveries have you had? {Please count stillbirths as well as live births}: _ _

How many cesarean deliveries have you had? {Please count stillbirths as well as live births}: _ _

How many of the deliveries resulted in a live birth? : _ _

How old were you at the time of your first live birth? age _ _

How old were you at the time of your last live birth? age _ _

Prenatal diagnosis ○ Yes (N°_____) ○ No If yes, result: _______

Modification of the disease after the pregnancy: ○ None ○ Worsening ○ Improvement

Menopause: ○ Yes ○ No If yes, physiological menopause: ○ Yes ○ No age _ _

Hormonal therapy: ○ Yes ○ No Modification of the disease: ○ None ○ Worsening ○ Improvement

Physical activity: Have you ever regularly played a sport? ○ Yes ○ No

If yes, report the two most played sports:

Sport (1): ________________________ ○ Professional ○ Amateur From age: _ _ to age _ _

Modification of the disease: ○ None ○ Worsening ○ Improvement

Sport (2): ________________________ ○ Professional ○ Amateur From age: _ _ to age _ _

Modification of the disease: ○ None ○ Worsening ○ Improvement

____

____

____

Physiokinesitherapy (PKT): ○ Yes ○ No

If yes, Duration of PKT treatment: From year: _ _ _ _ to year _ _ _ _

Modification of the disease: ○ None ○ Worsening ○ Improvement

Surgery: ○ Yes ○ No

If yes, operation (1): ________________________ year: _ _ _ _

Anesthesia: ○ General ○ Local ○ Epidural

Modification of the disease: ○ None ○ Worsening ○ Improvement

If yes, operation (2): ________________________ year: ______

Anesthesia: ○ General ○ Local ○ Epidural

Modification of the disease: ○ None ○ Worsening ○ Improvement

____

____

Family history *(information from at least three generations should be collected)*:

"Was/is any of your relatives wheelchair bound?"

"Did/does any of your relatives have a posture like yours?"

"Was any of your relatives sleeping with half-open eyes?"

Other considerations____

____

(pedigree attached)

Part B *NEUROLOGICAL EXAMINATION*

Age at Onset of motor impairment

Subjective age at onset (**when subject has noticed the appearance of motor impairment in his/her daily activities**):______________ years old

Site of muscle weakness reported by patient at onset

Muscle group:

Facial muscles: ○ Yes ○ No

Abdominal muscles: ○ Yes ○ No

Pelvic girdle muscles: ○ Yes ○ No

Asymmetry at onset: ○ Yes ○ No

Triggering events ○ Yes ○ No

Shoulder girdle muscles: ○ Yes ○ No

Distal lower limb muscles: ○ Yes ○ No

Distal upper limb muscles: ○ Yes ○ No

If yes, ○ Right ○ Left

If yes, event: (1) ________________________

(2) ________________________

Objective evaluation of age at onset by specific questions:

Have your relatives never noticed that you were sleeping with half-open eyes? ○ Yes ○ No

If yes, since age _______

Can you drink with a straw? ○ Yes ○ No

If no, since what age have you been unable to drink with a straw? _______

Can you to puff your cheeks? ○ Yes ○ No

If no, since what age have you been unable to puff your cheeks ?_______

Have you always been able to whistle? ○ Yes ○ No

If no, since age_______

Have you noticed the appearance of winged scapula? ○ Yes ○ No

If yes, since age _______

Have you ever noticed thinness of upper arms or a dropped shoulder? ○ Yes ○ No

If yes, since age _______

Have you ever noticed asymmetry of the mouth or smile when looking in a mirror or in past photographs from childhood? ○ Yes ○ No

If yes, since age _______

Other observations:____

Duration (years) from onset___________________________

Recurrent/chronic pain: ○ Yes ○ No If yes, since age _ _

Specify location________________

Precocious muscle fatigue during the common daily activities,before the onset of muscle impairment:
○ Yes ○ No If yes, since age _ _

Specify location________________

Other observations ○ Yes ○ No

____

Face:

Presence of:

Widened palpebral fissures: ..○ Yes ○ No

Puckered lips: ○ Yes ○ No

Horizontal smile: ○ Yes ○ No

Orbiculari oris hypokinesia during speech: ○ Yes ○ No

Dysartria: ○ Yes ○ No

Orbicularis oculi evaluation: ○ Normal (able to close heavily eyes)
○ Partial (able to close eyes but incapable to close heavily eyes)
○ Unable (unable to completely close eyes)

Ability to protrude lips: ○ Normal ○ Partial ○ Unable

Ability to puff out cheeks (against no resistance): ○ Normal ○ Partial ○ Unable

Asymmetric involvement of facial muscle: ○ Yes ○ No

if yes, specify side____

Scapular girdle:

Ability to abduct arms: ○ Whole (180°)
○ Complete but abnormal (patient can rise arms above head but only by flexing the elbow or using the accessory muscle)
○ Incomplete: >45° but <180° (specify if: ○ ≥90° or ○ <90°)
○ Incomplete: ≤45°

Pelvic girdle:

Ability to climb 4 stairs: ○ Without support
○ Without support but abnormally
○ With support (since age_ _)
○ Unable (since age_ _)

Ability to walk: ○ Without support
○ With support (since age_ _)
○ Unable (since age_ _)

Gait: ○ Normal ○ Waddling ○ Hyperlordotic ○ Steppage

Ability to stand up from a chair: ○ Without support
○ With support (since age _ _)
○ Unable (since age _ _)

Ability to rise from the floor: ○ Without support
○ With support (since age _ _)
○ Unable (since age _ _)

Use of wheelchair: ○ Not necessary ○ With manual control ○ With electric control ○ Bed bound

Legs:

Ability to walk on tiptoes and/or heels: ○ Normal ○ On tiptoes only ○ On heels only ○ Unable

Beevor's sign: ○ Positive ○ Negative

Part C

Medical Research Council (MRC) score:

Scores range from 0 to 5, with .5 increments (e.g. 3, 3.5, 4, 4.5, etc)

MUSCLE	***RIGHT MRC score***	***LEFT MRC score***	***ATROPHY Yes (right or left) /no***
Extrarotator muscles of upper limb*			
Triceps*			
Biceps*			
Common finger extensors*			
Wrist extensors*			
Long fingers flexors*			
Wrist flexors*			
Gluteus maximus			
Iliopsoas			
Biceps femoris			
Quadriceps			
Triceps surae			
Tibialis anterior			

(* *Muscles to be considered for FSHD score "Upper limbs involvement"*)

Strength of neck extensors muscles: MRC score___________

Weakness of pectoralis muscles: ○ Yes ○ No If yes, ○ Right ○ Left

Pectoralis muscles atrophy: ○ Yes ○ No If yes, ○ Right ○ Left

PRESENCE OF FOLLOWING TYPICAL FEATURES:

Scapular winging at rest: ○ Yes ○ No
(if yes, specify: ○ Symmetric winging, or ○ Asymmetric winging ○ > right; ○ > left)

Scapular winging on attempted shoulder abduction or forward flexion: ○ Yes ○ No
(if yes, specify: ○ Symmetric winging, or ○ Asymmetric winging ○ > right; ○ > left)

Horizontal clavicles: ○ Yes ○ No

Forward sloping of shoulders at rest: ○ Yes ○ No

Atrophy of pectoral muscles/ axillary creases: ○ Yes (○ > right; ○ > left) ○ No

Sunken or flattened appearance of the chest: ○ Yes ○ No

"Poly-hill sign" with neck, shoulders, and arms observed from behind in

fullest possible abduction (70–90°), with external rotation of the shoulders : ○ Yes ○ No

Hyperlordosis: ○ Yes ○ No

Orbiculari oris hypokinesia during speech: ○ Yes ○ No

PRESENCE OF UNCOMMON FEATURES:

Myotonic phenomenon: ○ Yes ○ No

Rippling phenomenon: ○ Yes ○ No

Eyelid ptosis: ○ Yes ○ No

Extra-ocular weakness: ○ Yes ○ No

Pharyngeal and lingual muscle weakness (persistent dysphagia): ○ Yes ○ No

Bent syndrome: ○ Yes ○ No

Early contractures: ○ Yes ○ No
(If yes, specify site____)

Dropped head: ○ Yes ○ No

Pes cavus: ○ Yes ○ No

Myoglobinuria: ○ Yes ○ No

Ogival palatus: ○ Yes ○ No

Others:____

Creatine phosphokinase (CPK) (value of two blood assays separated by at least one month):

○ Normal range

○ < 4x normal value (<1000 U/L)

○ > 4x normal value (>1000 U/L)

Instrumental evaluation

Cardiac involvement (ECG, echocardiogram):

Last ECG's report____ (date: _ _/_ _ /_ _ _ _)

Last echocardiogram's report____________________________________ (date: _ _/_ _ /_ _ _ _)

Electromyographic pattern of four limbs (detail the examinated muscles) (date: _ _/_ _ /_ _ _ _)

○ Myopathic pattern (○ Proximal ; ○ Distal)

○ Neurogenic pattern (○ Proximal ; ○ Distal)

○ Mixed pattern (○ Proximal ; ○ Distal)

Electroneurography of four limbs (detail the examinated nerves) (date: _ _/_ _ /_ _ _ _)

○ Normal ○ Abnormal

Report of last pulmonary function tests (FVC, MIP, MEP, Cough peak flow) (date_ _/_ _ /_ _ _ _):

____

____

Report of muscle biopsy (if available; please specify date and biopsied muscle)*: (date_ _/_ _ /_ _ _ _)

Biopsied muscle: ____

Report: ____

____

Other genetic test previously performed (if available):__________________________________

____

*(*please attach reports*)

Name of the Examiner: ___________________________

I - Facial weakness

0 - no weakness

1 - moderate weakness;

partial ability to do at least one of the following tasks:

- to close eyes
- to protrude lips
- to puff out cheeks

2 - severe weakness;

unable to do at least one of the following tasks:

- to close eyes
- to protrude lips
- to puff out cheeks

II - Scapular girdle involvement

0 - no involvement

1 - mild involvement with no limitation of arm abduction

2 - arm abduction > 45°

3 - arm abduction < 45°

III - Upper limbs involvement *

0 - no involvement

1 - at least two muscles affected with MRC >3

2 - at least two muscles with MRC ≤ 3

**The following 4 muscles will be assessed on each side: 1. triceps; 2. biceps; 3. common fnger extensors and wrist extensors; 4. long finger flexors and wrist extensors. Only the weaker muscles will be considered for evaluation.*

IV - Legs involvement

The ability to walk on tiptoes and heels will be assessed on each side:

0 - no involvement

1 - unable to walk on tiptoes or heels (only one task impaired)

2 - unable to walk on tiptoes and heels (two tasks impaired)

V - Pelvic girdle involvement

0 - no involvement

1 - able to walk and to climb stairs without support but abnormally/ because of posterior leg muscle hypotrophy

2 - able to walk unaided, to climb stairs or to stand up from a chair with support

3 - able to walk unaided but unable to stand up from a chair or to climb stairs without support/ more than 12 seconds

4 - able to walk with support

5 - wheelchair bound

VI - Abdominal muscle involvement

0 - no involvement

1 - presence of Beevor's sign

FSHD clinical score:________

	TYPICAL FEATURES	UNCOMMON FEATURES
**1. ONSET OF MUSCLE WEAKNESS**	☐Facial weakness of orbicularis oculi or oris<br>☐Scapular weakness with alterated ability to abduct arms<br>☐Humeral muscles (biceps/triceps)	☐Distal lower limbs onset with triceps surae weakness<br>☐Distal upper limbs onset<br>☐Pelvic girdle onset
**2. AXIAL MUSCLES INVOLVEMENT**	☐Hyperlordosis<br>☐Beevor's sign	☐Camptocormia<br>☐Dropped head
**3. FACIAL INVOLVEMENT**	☐Weakness of Orbicularis oculi (facial score ≥1)<br>☐Weakness of Orbiculari oris (facial score ≥1)	☐Weakness of extra-ocular muscles<br>☐Weakness of masticatory muscles (persistent dysphagia)
**4. SCAPULAR GIRDLE INVOLVEMENT**	☐Impairment of upper limb abduction with winged scapula or limitation of forward flexion (scapular FSHD score ≥1)	☐Isolated distal upper limb muscle weakness<br>☐Impairment of arms abduction (<90°) without winged scapula at rest and/or on attempted shoulder abduction or forward flexion
**5. PELVIC GIRDLE INVOLVEMENT**	------	☐Isolated and/or prevailing pelvic girdle muscle weakness
**6. LOWER LIMBS INVOLVEMENT**	☐Weakness of tibialis anterior muscles weakness	☐Early gastrocnemius and/or soleus atrophy/weakness
**7. BLOOD CPK LEVEL**<br>(at least two samples 1 month apart)	☐Normal range<br>☐< 4x normal value (<1000 U/L)	☐Value > 4x normal value (>1000 U/L)
**8. OTHER SIGNS**	☐Shoulders winging on attempted shoulder abduction or forward flexion<br>☐Horizontal clavicles<br>☐Forward sloping of the shoulders at rest<br>☐Sunken or flattened appearance of the chest<br>☐Atrophy of pectoralis muscles<br>☐Orbiculari oris hypokinesia during speech	☐Myotonic phenomenon<br>☐Rippling<br>☐Eyelid ptosis<br>☐Extra-ocular muscle weakness<br>☐Early muscle contractures<br>☐Cardiomyopathy<br>☐Early respiratory insufficiency (Non Invasive Ventilation, NIV; FSHD score <12)<br>☐Pes cavus<br>☐Myoglobinuria

Importantly: Indicate the presence of comorbidities / results of previous injuries / illnesses that can possibly affect the neurological examination:____
Extra-muscular involvement: ☐hearing loss, ☐epilepsy, ☐retinal involvement, ☐cognitive impairment

CATEGORY A

Category A1
Severe facial weakness (unable **both** to close eyes **and** to protrude lips) **+** impairment of upper limb abduction with winged scapula (scapular FSHD score ≥1) **+** absence of uncommon features

Category A2
Facial weakness (upper **and** lower facial weakness) **+** impairment of upper limb abduction with winged scapula (scapular FSHD score ≥1) **+** absence of uncommon features

Category A3
Facial weakness (upper **or** lower facial weakness) **+** impairment of upper limb abduction with winged scapula (scapular FSHD score ≥1) **+** absence of uncommon features

CATEGORY B

Category B1
Impairment of upper limb abduction with winged scapula (scapular FSHD score ≥1), no facial weakness + absence of uncommon features

Category B2
Facial weakness (facial FSHD score ≥1), no impairment of upper limb abduction + absence of uncommon features

CATEGORY C

Category C1
Subject with presence of at least one typical sign + FSHD score =0

Category C2
Subject without signs of muscle weakness **+** FSHD score =0

CATEGORY D

Category D1
Subject fulfilling criteria of categories A1, A2, A3, B1, B2 **+** at least one uncommon feature

Category D2
-Subject fulfilling criteria of categories C1 or C2 + at least one uncommon feature
-Subject no fulfilling criteria of any of the above categories
