# Supplementary material for: A novel clinical tool to classify facioscapulohumeral muscular dystrophy phenotypes
Source: J Neurol. 2016 Apr 28;263:1204–14. doi: 10.1007/s00415-016-8123-2 (PMC4893383; doi:10.1007/s00415-016-8123-2)
Supplement: Supplementary file 2 — Supplementary material 2 (PDF 51 kb) [file 415_2016_8123_MOESM2_ESM.pdf]

Supplementary table 1. Characteristics of the 56 FSHD patients enrolled in the inter-rater reliability study.

		Patients	
		**Number (n)**	**Percentage (%)**
**Sex**	*Male*	27	48.2
	Female	29	51.8
**Age at examination (years)**	*14-40*	19	33.9
	41-60	20	35.7
	61-74	17	30.4
**FSHD score**	*0-5*	28	50.0
	6-10	21	37.5
	11-15	7	12.5
**D4Z4 allele size (U)**	*1-3*	7	12.5
	4-6	38	67.9
	7-8	8	14.3
	9-10	3	5.4
